# Supplementary material for: Primary and hTERT-Transduced Mesothelioma-Associated Fibroblasts but Not Primary or hTERT-Transduced Mesothelial Cells Stimulate Growth of Human Mesothelioma Cells
Source: Cells. 2023 Aug 5;12(15):2006. doi: 10.3390/cells12152006 (PMC10417280; doi:10.3390/cells12152006)
Supplement: Supplementary file 1 [file cells-12-02006-s001.zip › Cells-2524949-R1 - PAGE images.pdf]

Original images of polyacrylamide gels (telomerase activity assay)

A

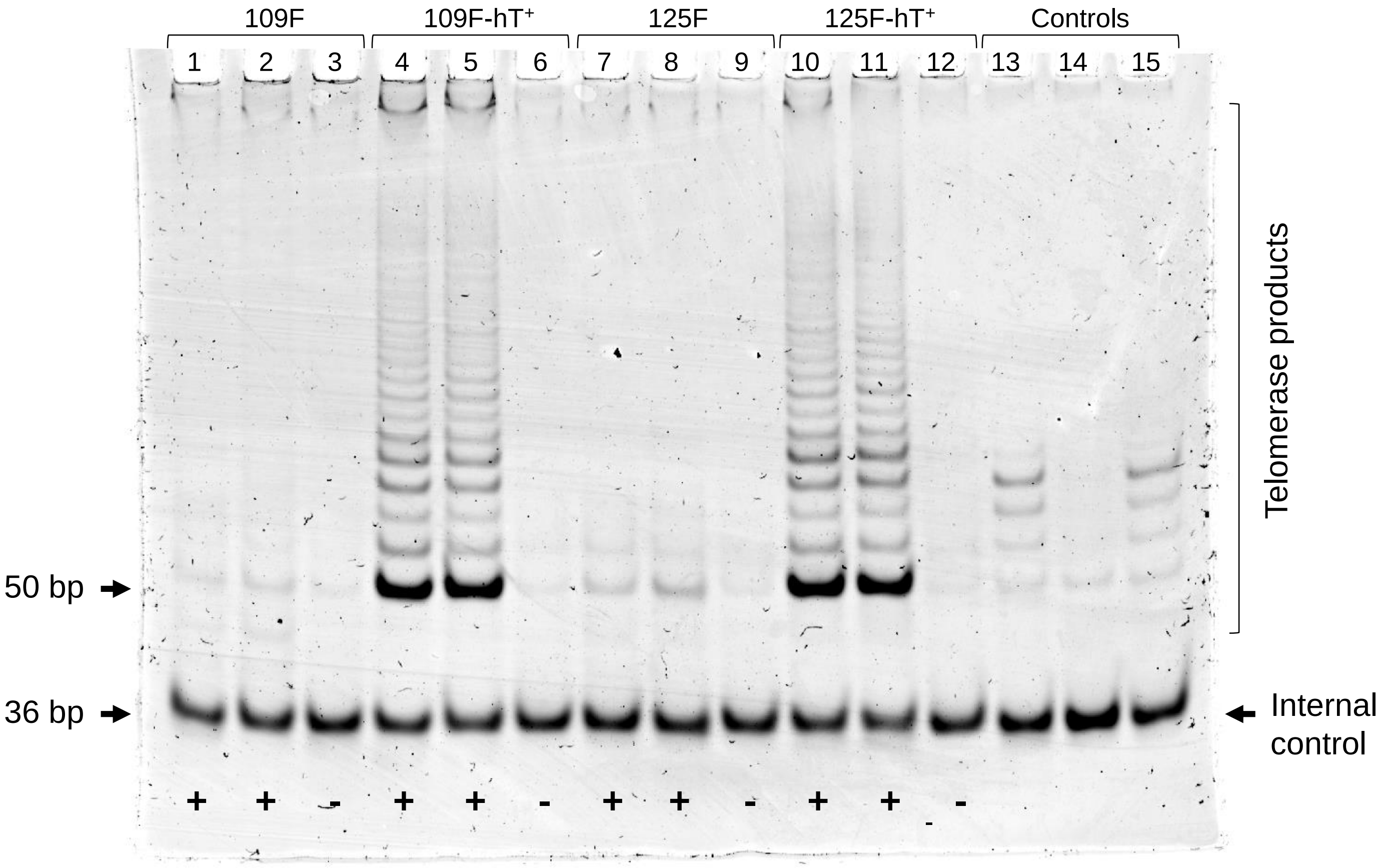

Lane 1-3: Meso109F (109F)
Lane 4-6: Meso109F-hT+ (109F-hT+)
Lane 7-9: Meso125F (125F)
Lane 10-12: Meso125F-hT+ (125F-hT+)
Lane 13, 15: TSR8 (≙ 16 TPG) [Positive Control]
Lane 14: Mere CHAPS buffer [Negative Control]
+ : non-denaturated probe
- : probe denaturated by heat

B

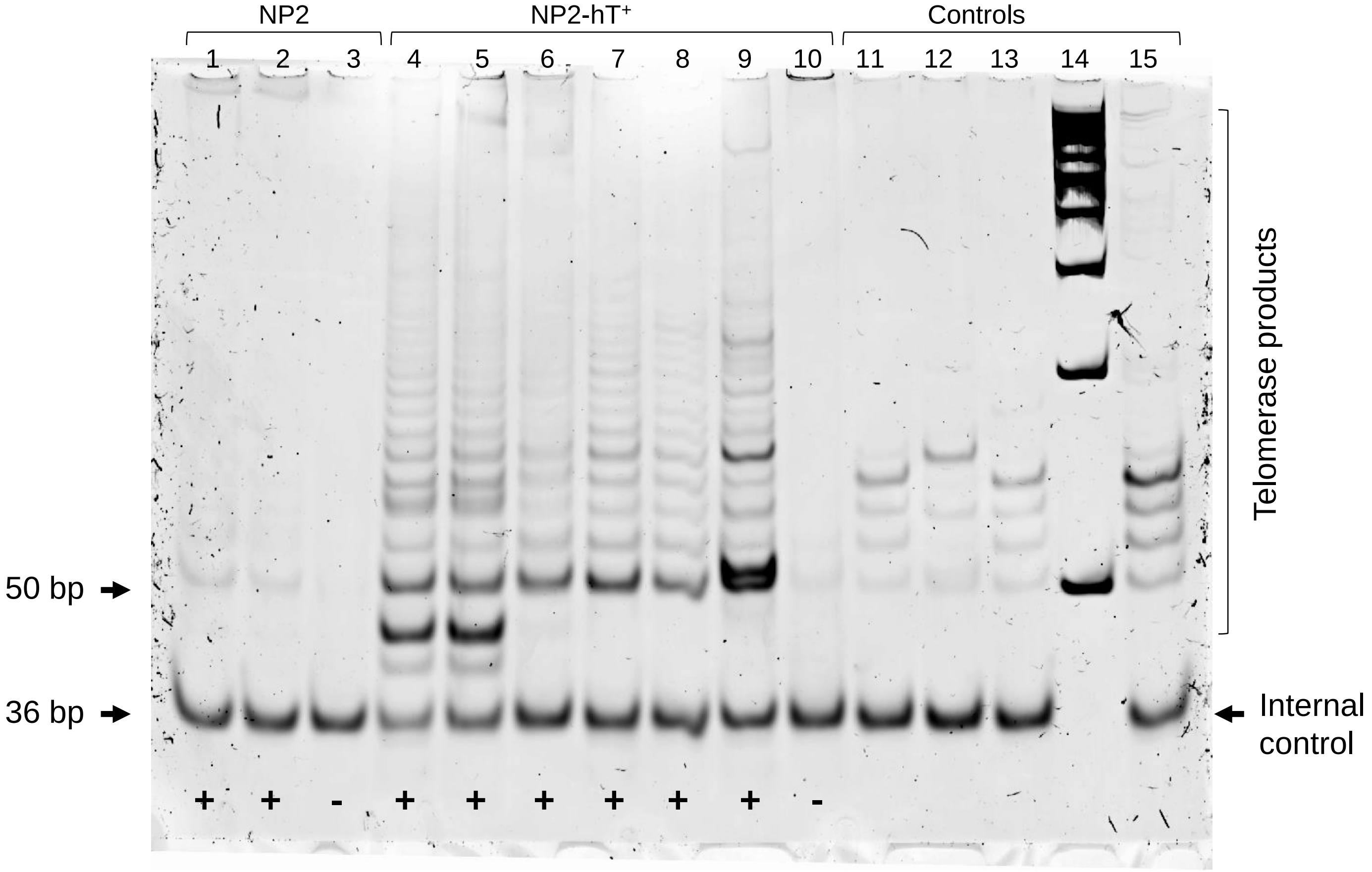

Lane 1-3: NP2 (NP2)
Lane 4, 5, 10: NP2-hT$^+$ (NP2-hT$^+$)
Lane 6, 7: NP2-hT$^+$ (NP2-hT$^+$) [1:5 dilution]
Lane 8, 9: NP2-hT$^+$ (NP2-hT$^+$) [1:10 dilution]
Lane 11, 13: TSR8 (≙ 16 TPG) [Positive Control]
Lane 12: Mere CHAPS buffer [Negative Control]
Lane 14: Marker (50 bp)
Lane 15: TSR8-D6 (≙ 64 TPG) [Positive Control]
+ : non-denaturated probe
- : probe denaturated by heat
