# Supplementary material for: Primary and hTERT-Transduced Mesothelioma-Associated Fibroblasts but Not Primary or hTERT-Transduced Mesothelial Cells Stimulate Growth of Human Mesothelioma Cells
Source: Cells. 2023 Aug 5;12(15):2006. doi: 10.3390/cells12152006 (PMC10417280; doi:10.3390/cells12152006)
Supplement: Supplementary file 1 [file cells-12-02006-s001.zip › Cells-2524949-R1 - Supplementary Figures clean version.pdf]

Supplementary Figures

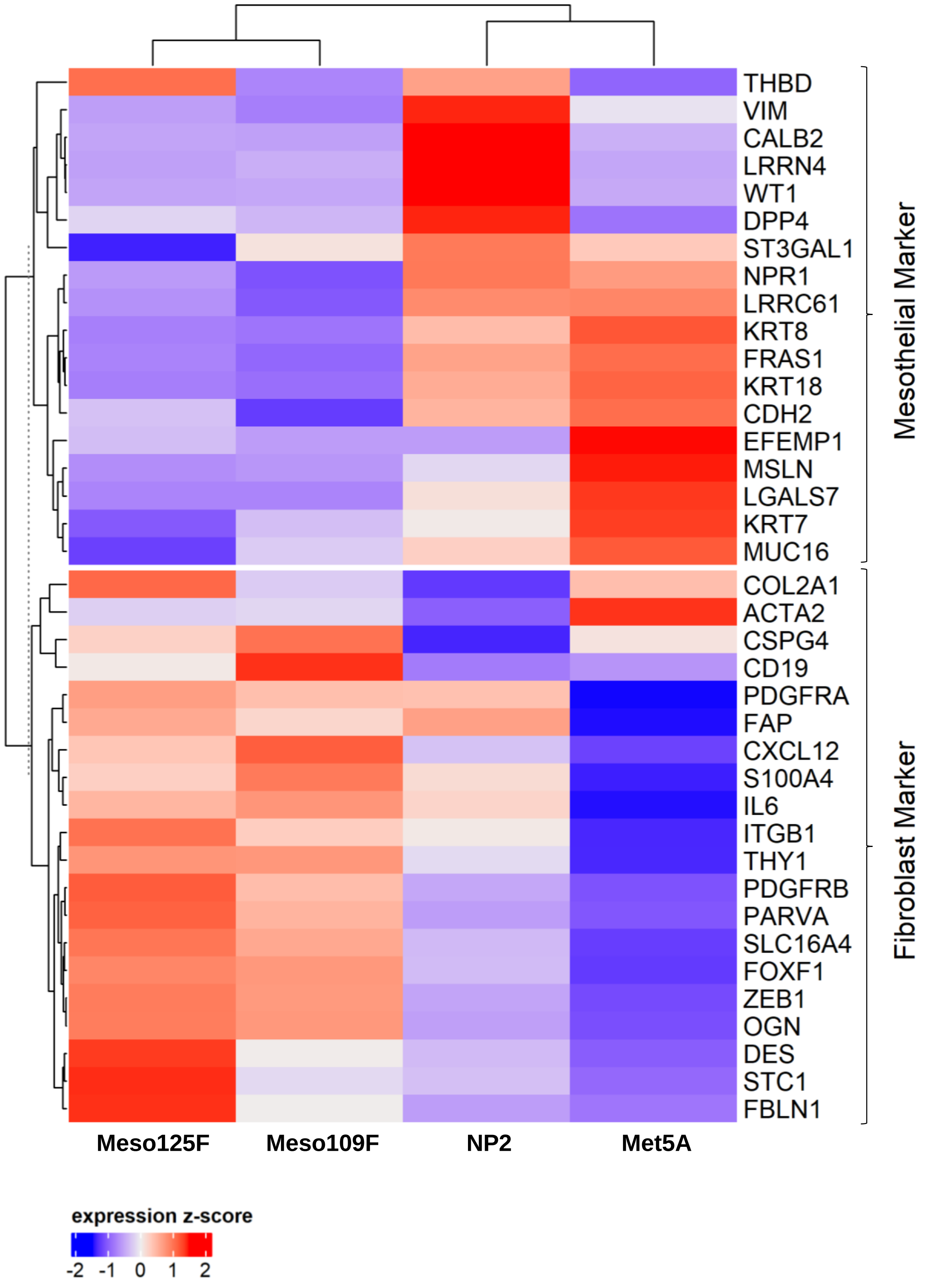

Supplementary Figure S1. Unsupervised clustering of mesothelial cells and Meso-CAFs. Transcriptomic data of mesothelial cells (NP2, Met5A) and Meso-CAFs (Meso109F, Meso125F) were subjected to unsupervised clustering using a list of common mesothelial and fibroblast marker genes.

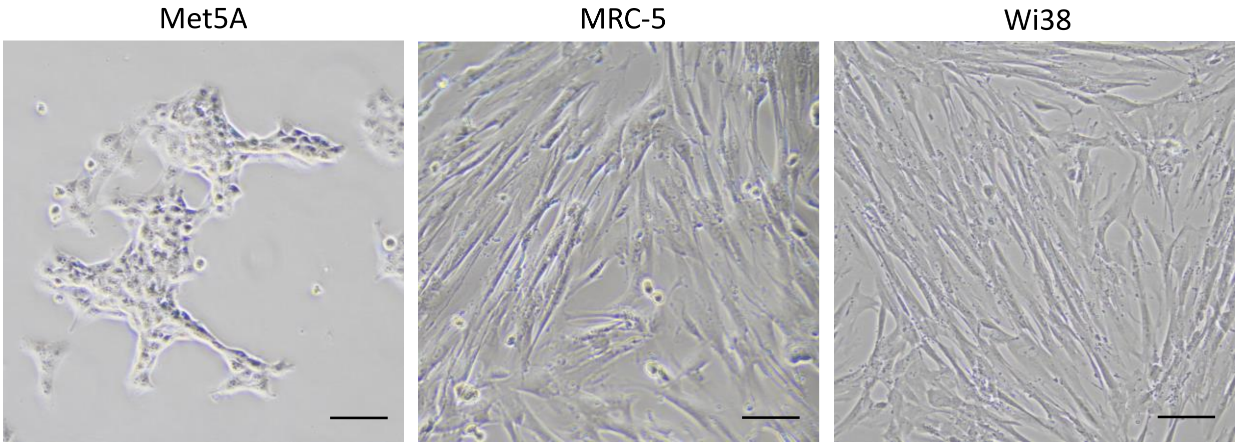

Supplementary Figure S2. Microscopic brightfield images of mesothelial cells (Met5A) and primary lung fibroblasts (MRC-5, Wi38). Scale bar = 100 µm (80x).

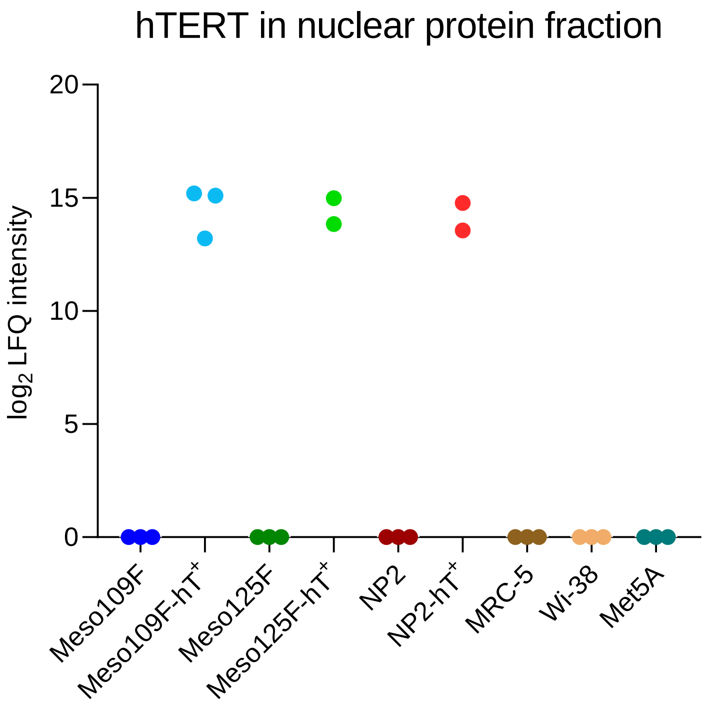

Supplementary Figure S3. Protein levels of hTERT in the nuclear protein fraction of the indicated cells. Each dot represents one biological replicate performed in duplicates.

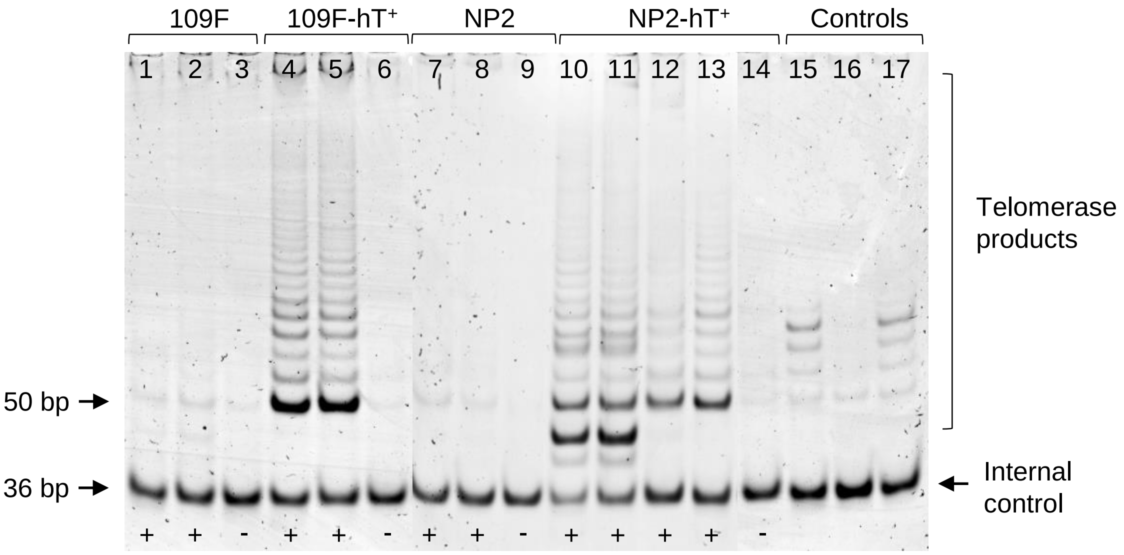

Supplementary Figure S4. Exemplary polyacrylamide gel image showing the telomerase activity of Meso109F (109F) [lane 1-3], Meso109F-hT$^+$ (109F-hT$^+$) [lane 4-6], NP2 (NP2) [lane 7-9] and NP2-hT$^+$ (NP2-hT$^+$) [lane 10-14] cells. Undiluted probes of NP2-hT$^+$ [lane 10, 11], NP2-hT$^+$ probes with 1:5 dilution [lane 12, 13]. Positive Control: Telomerase substrate oligonucleotide with eight telomeric repeats (TSR8) [lane 15, 17]. Negative Control: Mere CHAPS lysis buffer [lane 16]. + probes without denaturation, - probes denaturated by heat. Probes and TSR8 positive controls were subjected to the gel in duplicates.

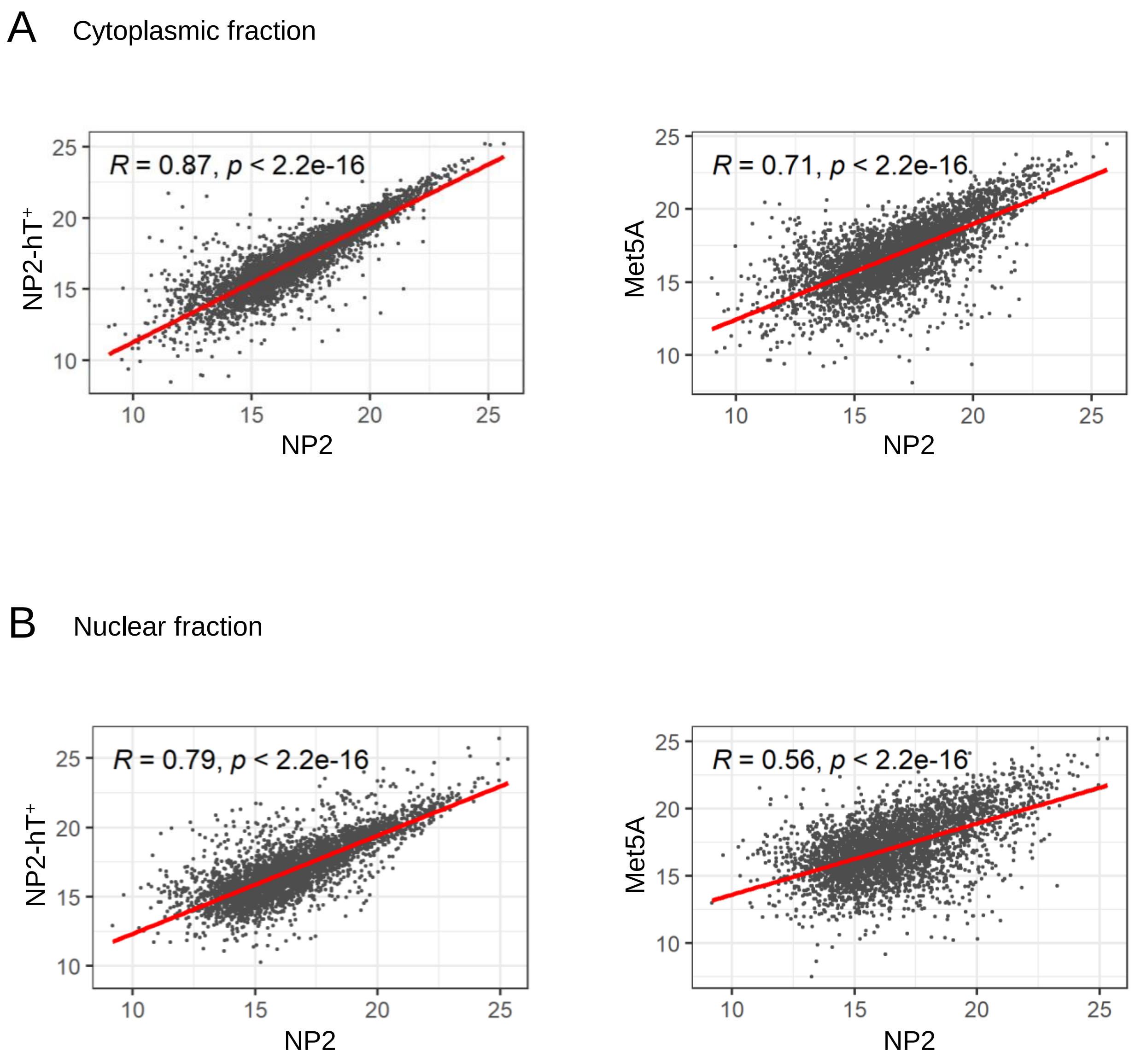

Supplementary Figure S5. Scatter plots of common (A) cytoplasmic and (B) nuclear proteins between primary NP2 and NP2-hT^{+} cells [left] and primary NP2 and Met5A cells [right]. Pearson correlation coefficients (R) and respective p-values are shown.

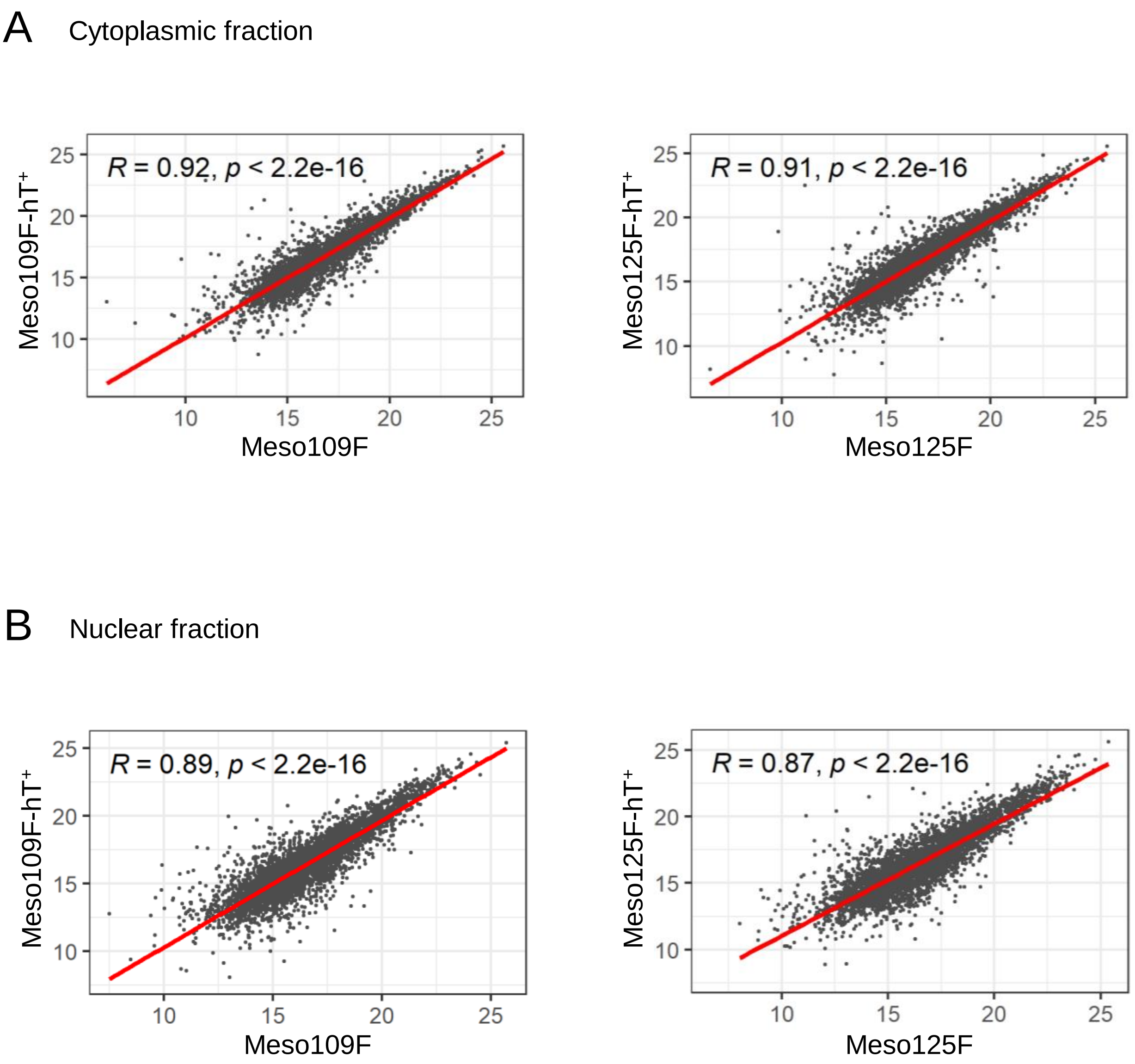

Supplementary Figure S6. Scatter plots of common (A) cytoplasmic and (B) nuclear proteins between the indicated primary and hTERT-transduced Meso-CAFs. Pearson correlation coefficients (R) and respective p-values are shown.

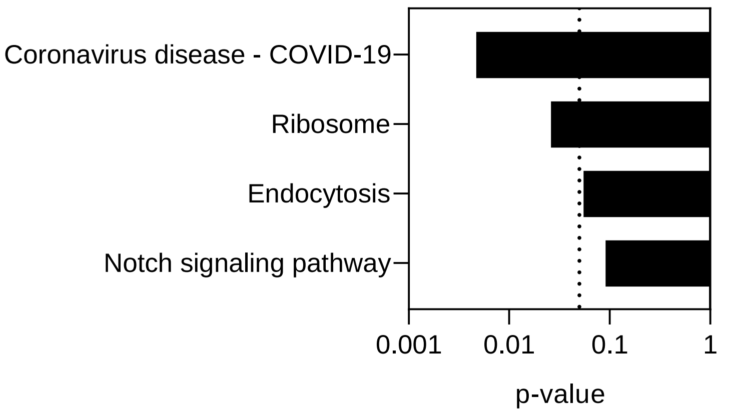

Supplementary Figure S7. Pathways associated with proteins commonly lost in hTERT-transduced Meso-CAFs (109F-hT$^+$, 125F-hT$^+$) compared to primary Meso-CAFs (109F, 125F) according to the KEGG pathway database. Due to the small number of proteins commonly gained in transduced Meso-CAFs compared to the primary cells, no analysis could be performed. A p-value of 0.05 is indicated with a dashed line.
